# Meeting of the Fulton County Medical Society

**Published:** 1915-04

**Authors:** R. R. Daly


### FULTON COUNTY MEDICAL SOCIETY.

Regular Meeting April 1, 1915.

By R. R. Daly, M. D.

Dr. Cosby Swanson presented a case of Syphilis with persistent mucous patches in the throat. He had two injections of Salvarsan without improvement and was given seven injections of venarsen, two a week, without any benefit. Later an equal number of doses of cacodylate of soda were administerd without changing the conditions. Venarsan is practically cacodylate of soda and this agent seemed to have no remedial action in the case presented.

Dr. E. P. Merritt said that in conjunction with Dr. Aven at the College clinic, he had administered venarsan many times. In a series of thirty-five cases four had not been helped, fourteen were improved and twenty-one showed excellent results. We should be careful in condemning venarsen too early. It is an American product and if it has any value, we should learn it and use it.

Dr. T. H. Smith presented a case of Mirror Writing appearing in a boy of 8 or 9 years. There is a negative history except that the child is left-handed and slightly hyperopic. He had little instruction in letters as a smaller child and no attention was paid to his learning to write until it was observed that he was writing his name in reverse. He wrote from right to left and slanted the letter from the left downward to the right. This, when turned around and placed before a mirror was fairly plain writing. It was really type writing, to use the accurate meaning of the words. He was soon taught to write the correct way and learned it without difficulty. He now writes either way with about equal facility. Literature shows about 50 cases of this peculiar condition. It is noted to be frequently associated with left-handedness, and comment is made as to imperfectly developed psychic relation. It may be that he doesn't learn to reverse retinal images properly.

Dr. Arch Smith said that his own brother when a child, had the habit of drawing things upside down, but there was nopsychic disturbance. Since then the man has become a successful architect and manages to get the cellars and roofs of his houses in the right relation.

Dr. R. R. Daly said that he had been questioning the child and its father and learned that the boy had had no instruction in writing except that he had imitated some older children whom he saw writing. He used his left hand and it was natural, without instruction, for him to start at the right hand side of the page and slant the other way. He learned to form some characters without knowing what they were. This, however, was not sufficient to account for his reversal to the type form. The boy is quiet, thoughtful and intelligent and talks slowly and well. His father replied that the child had never had any letter blocks except some small type blocks his older sister had used for stamping. He had played with these and learned to recognize letters in this way just as a printer does.

This at once accounted for the writing. He was merely recording his earliest impressions of letters. There is no psychic disturbance nor interruption in the normal activity of the visual act. It is not reasonable to suppose that there would be failure to see letters correctly and yet that he could establish proper relations with other matters in the world.

Mirror writing is always a childish affair. Children resort to it for amusement and in this instance by accident. Among older people a few use it as a simple trick to interest others. Many criminals have used it to conceal communications, and this, itself, shows the childish or undeveloped state of their minds.

The idea of reversals has attractive extensions. First, there is the curious matter of pallindromes where words and sentences read alike in reverse, for instance the following sign appeared in its proper place: REEB REGAL LAGER BEER. There are many examples of this and some of them are coincidents, while others are carefully invented.

Among criminals there are some who have learned to pronounce words backwards and have acquired so much fluency that their speech was rapid and incomprehensible to all but themselves. If one tries to work this out with a sentence containing words of one syllable only, he will soon note the cryptic character of the sound.

Dr. E. D. Highsmith reported a case of Substernal Goitre which he successfully removed, but which gave some peculiar symptoms. The man, a railroad conductor, was affected with much tremor and pains in the stomach and abdomen before operation. He stood the operation well, but three days later, he complained of severe pains in the right shoulder. These increased and became general and lasted many days. Eventually they were relieved and then he gained rapidly in weight and health. The question as to what caused the pains is unanswered.

Dr. Wells said that he had a similar case, only there was a longer interval before the pains began. They distorted the whole body for the time. Extensive elimination and rest gave her relief.

Dr. G. D. Ayer said that he had recently removed the tonsils in two cases of exopthalmic goitre and that symptoms of hyperthyroidism subsided in marked degree.

Dr. Hansell Crenshaw said that many cases of cardiac pain were pseudoangina due to toxins, such as might come from diseased tonsils. Operative procedure is indicated in all these and in each case of goitre presenting extensive toxicity.

Highsmith said that he had not noted the tonsils in his case before the goitre held his attention. As to the cause of goitre, there is nothing new to suggest except that it seems to be increasing around the great lakes. The Alpine conditions are well known.

Dr. J. S. Derr related a case of right sided colic in the kidney. X-Ray examination showed no stone, but a shadow in the true pelvis. Later this mass was larger and as big as an orange. Pelvic examination confirmed skiagram. Operation displayed tumor. It was suggested that it was a small ectopic pregnancy, but investigation showed it to be a hematoma of the right ovary. Plates were shown.

Dr. M. T. Davis read a paper, "Cardiac Hypertrophy and Dilatation." It appears elsewhere in The Journal.

Dr. H. R. Donaldson said that the treatment of any case of heart disease was always complicated by the character of the digitalis one gets. The standardized preparations are not always secured by the patient in home matters and it is incumbent upon the doctor to care for this.

Dr. E. C. Thrash said that he doesn't know what "palpitation" is. The word is vague and undefined; it covers all the arhythmias, double systole and weak and strong systole. They are symptomatic and organic and are due to various conditions of the ventricles or the large size of the heart. The term "palpitation" should be discarded.

Dr. R. T. Dorsey said that hypertrophy and dilatation are not always present together and told of specimen in McGill University showing this. Aortic stenosis may not have dilatation at all, but it will show hypertrophy. Dilatation is not always permanent, for there is manifest transient dilatation upon excessive exertion.

Dr. G. M. Niles said that incidentally to his work upon the stomach, he had during the last 14 months, seen 600 hearts with the fluoroscope and he had taken note of them. Only a few had come for heart trouble. The conformation of the heart varies with the individual just as do the parts of the man with which we are more familiar. Moreover, the normal picture varies considerably from the standard in the books. Men of 35 or 40 who have been athletic have large round hearts, while other men may have enlargements that are more angular and broader. In people of 60 to 70 the heart appears small and long. It may be that this is a type of senile heart. These things have not been spoken or written of so far as he knows.

Dr. C. C. Aven said that he had a patient with the apex in the seventh space and no indication of hypertrophy. There was increased dullness over all the area. Four months later the dullness and apex were in normal locations, showing the dilated heart had grown small again. Digitalis trouble can be avoided, he thinks, by using the powdered leaves.

Davis closed saying that he agreed with Aven as to digitalis leaves and added that the English leaves were the best to be had. Niles' work with the X-Ray was illuminating and he hoped to hear more about it.

Dr. George H. Noble read a paper on the "Dynamics of Displacements of the Uterus Downward and Backward."

It was illustrated with charts showing just how the various pressures could be applied and measured and how the deformities were likely to follow abnormal anatomical conditions. The experimentation had been done by introducing a rubber bag within the sack of an umbilical hernia and another within the vagina. These were to be filled with air and were connected with registering apparatus showing the pressures under various conditions.

Dr. J. F. Denton said that since the anatomical conditions were supposed not to be extensively changed in virgins, he had not been able to understand just how Noble's dynamic principles applied to them.

Dr. W. F. Shallenberger said that though cardinal ligaments might hold the womb in place by themselves for a time, the intact perinaeum is the strongest force supporting the viscera of the pelvis and abdomen. The utero-sacral ligaments hold the cervix in hollow of the sacrum; when they fail, the cervix sags forward and the abdominal counter-force no longer holds. Prolapse even in virgins may follow. In many prolapsed cases there is hypertrophied cervix which adds to the trouble. Retroversion after pregnancy should be instantly remedied. It causes sub-involution and adds to downward pull.

Dr. J. W. Duncan said he had a lady who suffered with profuse menstruation for several days and then presented complete procidentia. The womb was pushed back but would not stay. He asked what to do with such a case and whether immediate operation was indicated.

Dr. G. W. Quillian raised the question as to athletics in young women from 18 to 24. Could displacements result from such activities?

Dr. L. Sage Hardin said that there are so many fundamental disturbances in displacements that many other conditions coincide. Thyroid and other toxic states are conspicuous illustrations of this. As to faulty development in virgins, the assumption of the knee chest position in calisthenics might be of value in training girls.

Noble closed and said that Denton's question was a large one. Young women have lack of development, thyroidism, slight invalidism and painful menstruation. The round ligaments are frequently as small as the uterus itself. There is much loose tissue around the cervix and this allows it to drop. As to Duncan's case, some form of repair operation is indicated. Pressure of supports would likely do great injury to tissues already stressed.

Dr. E. Bates Block read a paper, "The Muscular Layer of the Perineurium."

This is an original investigation showing that there are unstriated muscular fibres in the perineurium. They have a long nucleus and stain exactly as do such fibers elsewhere.

This is of great importance in physiologic phenomena as well as in diseased conditions of the nerves. We do not have a lymph heart as do the lower animals and there is no apparent cause for the lymph circulation at the nerves except this layer. Hysteria is of sympathetic origin. Just how hysterical paralysis occurs has not hitherto been explained, but the influence of these muscular fibers would account for it.

Dr. C. W. Gould said that his attention had never been called to this matter before. He doesn't see just how the muscular fibers can be demonstrated but if they are there, it is a great thing in the history of medicine to have found them.

Dr. A. H. Bunce said that he had seen the slides and specimens and they were fine. He can't discuss them well because the matter is all so new, but the reasoning is good and the subject so enticing as to lead to further study of it.

Dr. R. T. Dorsey said it was exceedingly interesting and he was thinking of the new fields of study and activity it would open if it proved true of all the nerve fibers.

Dr. E. C. Thrash said that lymph circulation in this way was not logical. There are no valves in the lymphatics at the nerves and nothing to determine the direction of the circulation even if the muscles did act as Block thinks. There should be some other than the microscopic test to prove the presence of the muscles.

Dr. Arch Smith said that tetanus toxin travels along the nerves and the muscles may cause this movement.

Dr. Hansell Crenshaw said that the thesis was interesting and logical. Whether hysteria is a disease of the sympathetic nervous system is an open question. Contracture of a voluntary muscle group as in finger and hand is hard to account for on this basis.

Block closed. He said that he had made many more tests and stains than he had related and he had subjected his slides and thesis to competent laboratory men. All go to show the difference between the perineurium and connective tissue. The morphologic difference in the nucleii is the most important. The perineurium looks just like the muscular coat of the blood vessels.

### REGULAR MEETING APRIL 15, 1915.

Dr. L. W. Childs presented a specimen of Persistent Urachus that he had recently removed from a young Greek. The man had been shot during 1912 in the abdomen, but there had been no intestinal wound. About a year later the bullet had come to the surface near the spine of the ilium. Some time after this he complained of a tumor and bad secretion coming from the navel. This was probed and then injected with methyl blue and subsequently removed. It proved to be a persistent urachus. Recovery.

Dr. Allan Bunce said that hitherto there had been difficulty in Gram stains because of lack of permanency in the coloring and in the reagents. He presented a formula of a permanent stain and showed a bottle that he had been keeping for over a year with the reagent in good condition. The formulæ are: 1. 2% Crystal violet in C. P. Methyl alcohol; II. 2% Saffranin in distilled water.

He also showed Keidel's apparatus for taking blood samples at the bedside if desirable and always keeping them sterile.

Dr. E. Bates Block presented a case of Habit Spasm in a young woman. About the summer of 1913 she was observed to be shaking and to have convulsive actions of the arms. There was no loss of consciousness. The spasmodic activities became more general until they involved the whole body and made it impossible for her to eat solid food or to sleep for more than an hour at a time.

Her history is unimportant except that she was said to have had mumps in 1914. This is doubtful. There has been no trouble with the tonsils or rheumatism. The spasms have been exceedingly violent and so marked that it was painful to see them.

Wasserman is 4 plus. She had measles in childhood with the left eyelid drooping for nearly a year afterward.

Prior to Block's seeing her she had had fever for a year, sometimes as much as 101. Her thirst was inordinate and she drank several quarts of water each night because of dryness of the mouth. She was placed upon arsenical and mercurial treatment and grew much better so that she is able to be before the Society and shows no spasm, but there is a swelling and a sinus upon her right cheek from which at present a clear secretion without bacteria comes. It has been there a long time and at first showed pus and staphalococci, A. An X-Ray showed erosion of lower jaw and fracture at the angle and erosion of the upper jaw. The intense thirst and dryness are judged to be due to closure of Steno's duct on the right side. She now drinks two gallons of water a day for the great dryness and of course, there is corresponding frequency of urination.

Dr. S. R. Roberts asked if this were not a true Xerostomia.

Block replied that in his opinion it was due entirely to the blocking of the parotid.

Dr. E. C. Thrash read a paper, "Injection of Alcohol into the Superior Laryngeal Nerve for Painful Tubercular Laryngitis."

He spoke of the great suffering such patients have to endure and said that anything that would give them relief, even if it did not cure them was of the utmost importance.

Tubercular Laryngitis is not primary; it comes from disease of the lungs and when it does appear and extend beyond the cords themselves, it is likely to be in the last stages of the life of the patient. Thus far no local treatment has proved of avail and they could neither talk nor breathe without pain, while swallowing often became impossible. He finds the internal carotid with the tip of his finger and then presses inward and backward until he finds the space between the hyoid and the thyroid cartilage on that side. Pushing the carotid as far aside as it will go, he is thus protected from injuring it and has a clear field in which to thrust the needle of the syringe. This is pushed directly in until it is felt to snap through the thyro-hyoid membrane and then a small amount of a weak solution of cocaine is injected and the needle gradually withdrawn so that the cocaine is spread over the line of injection. Then from a half to a whole cubic centimeter of alcohol is injected at the same place beneath the thyro-hyoid membrane. It is not claimed that the trunk of the nerve is reached and pierced. It would be absurd to think that anyone could do that. But the alcohol will spread in its effects over half an inch of space and that is sufficient to reach and anesthetise the nerve. Care must be taken not to pierce the thyroid cartilage nor to get into the tracheal wall because of the risk of emphysema, but this caution is easy to maintain. Both sides may be injected at the same sitting. The relief comes very quickly and patients who could not swallow or breathe without agony will call for food and enjoy a return to it. The time of freedom from pain varies with the patient from two weeks to two months. The injections are to be repeated as soon as pain returns. They are as easy to make as an intravenous injection. The pain will sometimes go to the ear and teeth for a few minutes when the nerve is reached.

Dr. Allan Bunce said that he had seen this work done and some striking results follow immediately.

Dr. Arch Elkin said that he had seen several of Thrash's cases and that there was no doubt as to the simplicity and efficacy of the measure.

Dr. Hansell Crenshaw read a paper, "Diagnostic Significance of Tremors." He described, classified and grouped Tremors as they are seen clinically in nervous diseases and degenerative changes and indicated their general and minute significance. He made one appropriate and apt change in nomenclature when he said that "intention" tremor was almost a misnomer because there was no preconcept in regard to it. He called it an "effort" tremor and placed in contradistinction to passive tremors.

He showed tracings taken from passive tremors where he had been able to get them and measure them. The records were enlarged photographically and the waves indicating oscillations varying from three or four a second up to ten or twelve a second were shown. He stated that as yet he had not been able to devise apparatus for recording effort tremors, but hoped to present such a machine at a subsequent meeting.

Dr. E. Bates Block commented favorably upon the paper and praised the records shown.

Dr. Robin Adair read a paper, "Pyorrhoea Alveolaris With Special Reference to the Emetine Treatment."

He took issue with Bass and others as to the etiology of the endamoeba buccalis and said that the disease could not be cured by killing them with emetine. The disease is fundamentally a surgical one and if the pus cavities are cleansed thoroughly from a surgical point of view and then sealed so as to permit them to unite their walls either by collapse or healthy granulation, the disease will be cured. And if this is not done, the disease will persist no matter what amount of emetine is given. He read letters from prominent dentists over the country in support of his thesis.

Dr. G. M. Niles said that if he were to start a farm, he would go to a practical and skilled agriculturalist for advice and by the same token, he listens with respect to what Adair has to say on this matter. Adair has been successful for many years in the treatment of Pyorrhoea and he knows what he is talking about. Just after Bass' article first came out, he had a case of pellagra complicated with pyorrhoea alveolaris. He gave him emetine for six successive days and he improved, but he sent the man to a dentist later and secured further improvement through the surgical attention. He has had two other similar cases. None has gone to Adair because they were out of town cases and already had good family dentists. Emetine is a new proposition and we must use care in following the pendulum that just now is swinging its way. We can not get along without good dentists.

Dr. E. C. Thrash said that he agreed with Niles that the dentists should attend to these cases, but there was no question from the bacteriologist's standpoint as to the presence of the endamoeba. He has examined many cases of pyorrhoea and always found that Emetine has an influence upon them unquestionably, but where there has been bone destruction the drug is inefficient and the surgical treatment is imperative. Thrash said that he had had bleeding gums for a long time himself, and they were cured by the use of emetine.

Dr. S. T. Barnett said there should be an increasing degree of co-operation between the dentist and the physician and that mouth hygiene should be in the mind of every internist and of every surgeon for that matter. He told of a case of severe Bright's disease that was sent to him from a neighboring state. He thought the man was going to die in a few days. He had a very foul mouth and Adair treated this so well that the man improved generally and became well enough to go home. He asked what preparation Adair used with which to seal the cavities after they had been treated for the pyorrhea.

Adair said that emetine does have a haemostatic influence and is of value. It doubtless has its claimed effect upon the endamoeba too, but that doesn't mean all it is claimed as to destruction of the peridental membrane. The statement that 95% of adults have pyorrhoea is not borne out by his experience and observation. Certainly not that many people who come to his office have it. In answer to Barnett, he said that he had two solutions. After the pockets were thoroughly cleansed they were treated with No. 1, which was a mixture of iodine crystals and creosote. This was used for as many days as were necessary to the complete sterilization of the pockets. They were sealed with No. 2, which was super-saturated tannate of glycerin. It was applied when the parts were dry and then the saliva was allowed to touch it. Instantly an impermeable seal was formed which could not be torn away from the gums until after 48 hours.

Dr. O. O. Fleming related a case of imperforate rectum, which he operated upon in a six days old baby. He was able to make a sphincter with fibres found and the child did well for ten days, when it died of intercurrent pneumonia.

